# The PitNET Puzzle: From Zero to Linking Molecular Behavior with Neurosurgical Aspects

**DOI:** 10.3390/medicina61111973

**Published:** 2025-11-03

**Authors:** Amira Kamel, Ligia Gabriela Tataranu, Bianca-Cristina Cristutiu, Anica Dricu, Radu Eugen Rizea

**Affiliations:** 1 Neurosurgical Department, Bagdasar-Arseni Clinical Emergency Hospital, 041915 Bucharest, Romania; kamel.amyra@yahoo.com (A.K.); bianca.cristutiu@gmail.com (B.-C.C.); radu.rizea@umfcd.ro (R.E.R.); 2 Neurosurgical Department, Carol Davila University of Medicine and Pharmacy, 020022 Bucharest, Romania; 3 Biochemistry Department, Carol Davila University of Medicine and Pharmacy, 020022 Bucharest, Romania; anica.dricu@live.co.uk

**Keywords:** pituitary gland, pituitary neuroendocrine tumors, biomolecular, neurosurgery

## Abstract

The pituitary gland is considered the conductor of the hormonal orchestra, and despite its small dimensions, numerous tumoral lesions can arise within it. Over the past decade, substantial changes have been made regarding the nomenclature, which are summarized in the 5th Edition of the World Health Organization Classification of Endocrine and Neuroendocrine Tumors. Furthermore, significant breakthroughs in biomolecular mechanisms have been uncovered, which have formed the basis for the new classification. The management of these lesions varies according to several factors such as tumoral dimensions, hormonal activity, symptomatology, and radiological findings. At the same time, the therapeutic goal is represented by normalization of hormonal hypersecretion if present, control of tumoral growth and/or relief of mass effect symptoms, and preservation or restoration of the pituitary function. The current narrative review aims to explore the link between biomolecular aspects, the extent of resectability, and the postoperative outcome.

## 1. A Brief Overview

### 1.1. Epidemiological Aspects

Out of all intracranial tumors in the adult population, pituitary neuroendocrine tumors (PitNETs) account for approximately 15% as per 2020 [1]. Earlier studies have shown that the estimated prevalence of these lesions in postmortem studies was 14.4% compared to 22.5% in radiography studies. The overall prevalence for both groups is estimated to be 16.7% [2]. Although epidemiologic studies have been used to estimate the current prevalence, their significant limitations are represented by dependence on population-specific registries and clinical diagnosis, which excludes silent incidental tumors [2]. Immunohistochemistry analyses have shown that, among all PitNETs, approximately 43% are prolactinomas, 2.8% somatotropinomas, 4.9% corticotropinomas, 1.4% gonadotropinomas, and 0.7% thyrotropinomas [2]. Functioning PitNETs, especially prolactinomas, have a clear female predominance, particularly in reproductive-age women, while non-functioning PitNETs have a male predominance and tend to be diagnosed later, at larger sizes [3]. Non-functioning PitNETs are the most common, while in functioning PitNETs, the most encountered tumors were represented by prolactinomas, somatotropinomas, corticotropinomas, and thyrotropinomas [4].

### 1.2. The Controversies of Nomenclature

In 1932, Harvey Cushing first introduced the term “pituitary adenoma” to describe the underlying cause of acromegaly. Notwithstanding that these tumors were subsequently treated as benign lesions, various characteristics differentiate them from typical benign-featured tumors [5]. These specific differentiating features were not only represented by a propensity for hemorrhage and necrosis, but also by the frequent invasive behavior [5].

As previously stated, pituitary tumors can invade the surrounding anatomical structures and grow rapidly despite optimized therapeutic management, and in these cases, the lesions are considered aggressive [6]. However, it is worth mentioning that invasiveness does not necessarily correlate with aggressiveness [6].

The latest changes in nomenclature were proposed in 2022 and were summarized in the 5th Edition of the World Health Organization (WHO) Classification of Endocrine and Neuroendocrine Tumors [7]. This classification introduces significant biomolecular changes, characterizing tumors beyond the conventional hormonal activity that has previously been the basis. Nowadays, tumoral lesions are classified based on cell lineage as determined by expression of transcription factors, hormones, and other biomarkers [7]. Although most scientists and physicians agreed on the new classification, others pleaded for reconsideration. The most cited reasons against the new nomenclature were represented by a more sinister connotation while removing information regarding developmental origins, a lack of distinction between endocrine and neuroendocrine cells, as well as the controversies regarding whether similar terminology should be attributed to other endocrine organs’ neoplasms [8]. Furthermore, the term PitNET asserts high-risk behavior, which is an infrequent exception [8]. The psychological burden of patients with benign pituitary lesions diagnosed as PitNET could lead to unjustified anxiety and result in more aggressive therapeutic management, even in cases of asymptomatic microadenomas [9]. However, despite ongoing debates, the new classification has been widely accepted and incorporated into clinical practice, as it raises the opportunity to implement structured and detailed reporting of these lesions [10,11]. Nevertheless, despite significant advances, especially in the biomolecular field, in most cases of PitNETs, the mechanisms behind pathogenesis are yet to be fully uncovered, and remain enigmatic in the majority of cases [12].

### 1.3. How to Diagnose a PitNET?

Based on their dimensions, pituitary tumors are classified into microadenomas (<10 mm), macroadenomas (≥10 mm), and giant adenomas (≥40 mm) [13]. In extensive lesions, compression on the neighboring anatomical structures can lead to various conditions like hypopituitarism, chiasmal syndrome, or diabetes insipidus. Thus, in cases where the neuroimaging examinations show tumor impingement, visual field testing and an evaluation for hypopituitarism are indicated [13]. In many instances of compression on the pituitary stalk, hyperprolactinemia can appear, known as the stalk effect. This phenomenon is due to pituitary lactotroph disinhibition. However, the stalk effect is temporary, as it resolves with tumor excision, and the prolactin level will drop postoperatively [14].

Some PitNETs can secrete excessive hormones and are known as functioning, while between 22% and 54% are not associated with clinical evidence of hormonal hypersecretion, and are called non-functioning [15].

**Figure 1:**
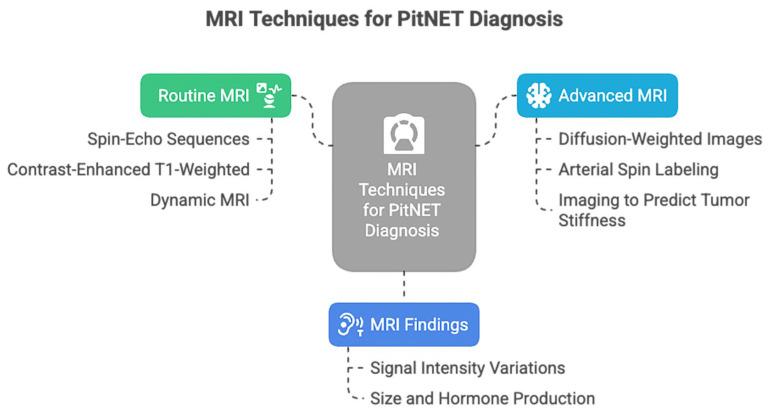
MRI techniques used for PitNET diagnosis.

Regarding the neuroimaging diagnosis, magnetic resonance imaging (MRI) is the most helpful imaging modality of choice (Figure 1).

The routine MRI, represented by non-contrast or contrast-enhanced MRI, is useful for initial screening and lesion detection [5]. Dynamic MRI is also considered a valuable diagnostic tool in PitNETs, facilitating the detection of microadenomas and the visualization of compression and displacement of anatomical structures by macroadenomas. Furthermore, dynamic MRI could be used to predict GH-producing tumors in PitNETs [16]. However, nowadays, even more complex techniques are available, such as diffusion-weighted images (DWI), which reduce artifacts and enhance the image quality of the sellar region, supporting a precise detection of microadenomas [17]. Perfusion imaging can assess tumoral vascularity, can predict hemorrhage risk [18], and could monitor the response to medical therapy [19].

The stiffness of PitNETs can impede a complete resection, especially when using the transsphenoidal approach; thus, it could be helpful for surgical planning to predict this tumoral characteristic. Although no current imaging technology can fully evaluate the preoperative viscoelastic consistency of PitNETs, magnetic resonance elastography (MRE) can measure the propagation of mechanically induced shear waves through tissue to calculate stiffness [20,21].

Although certain assessment methods are highly suggestive, a multidisciplinary team is essential to establish a detailed and definitive diagnosis, which serves as the foundation for choosing the optimal treatment option.

### 1.4. Therapeutic Management

The therapeutic management of PitNETs varies according to several factors, including tumor dimensions, hormonal activity, symptomatology, and radiological findings [22]. The primary goals of therapeutic management are the normalization of hormonal hypersecretion, if present, control of tumor growth and/or relief of mass effect symptoms, and preservation or restoration of pituitary function. In functioning PitNETs, the therapeutic approach depends on the tumor subtype [23], and details about each type will be further discussed.

## 2. From Genes to Neurosurgical Margins

PitNETs originate from specific cells in the anterior hypophysis and are currently characterized by the expression of transcription factors, according to the 5th Edition of the WHO Classification of Endocrine and Neuroendocrine Tumors (Table 1) [12].

**Figure 2:**
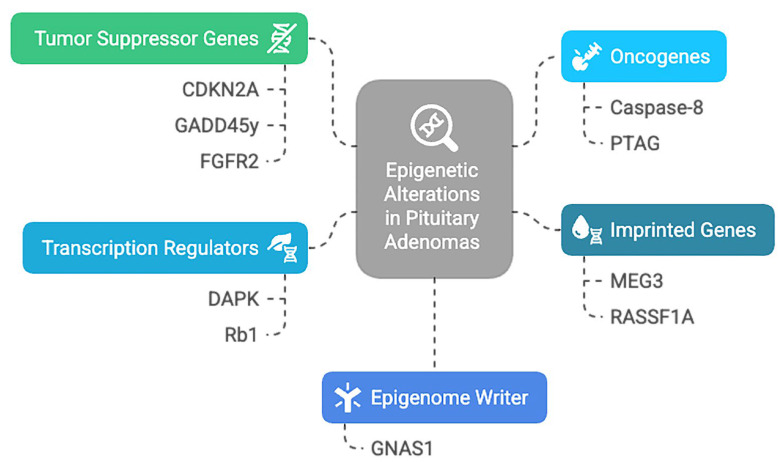
Epigenetic alteration encountered in PitNETs: CDKN2A—Cyclin-Dependent Kinase Inhibitor 2A; GADD45y—Growth Arrest And DNA Damage Inducible Gamma; FGFR2—Fibroblast Growth Factor Receptor 2; PTAG—Rhomboid Domain-Containing Protein 3; DAPK—Death Associated Protein Kinase 1; Rb1—RB Transcriptional Corepressor 1; GNAS1—Guanine Nucleotide Binding Protein (G Protein), Alpha Stimulating Activity Polypeptide 1; MEG3—Maternally Expressed 3; RASSF1A—Ras Association Domain Family Member 1.

The biomolecular mechanisms underlying pituitary tumorigenesis are intricate and are still being studied. It is worth mentioning that the epigenetically mediated gene dysregulation represents a frequently encountered characteristic in tumoral development. Increasing evidence suggests that a significant contribution comes from bone morphogenic protein (BMP), Wnt, and fibroblast growth factor (FGF) families [24]. In addition, current evidence supports epigenetic alteration in other various PitNET genes, categorized based on function and alteration [25], as summarized in Figure 2.

It has been stated that the DNA methyltransferases play significant roles as oncogenic factors in PitNETs development and progression. The overexpression of DNMT proteins is associated with macroadenomas, invasiveness, and aggressiveness, suggesting a poor prognosis, regardless of the therapeutic management [26].

Recent studies have revealed clinically relevant epigenetic markers that can aid in diagnosing and prognosticating patients with PitNETs, as serum and plasma cell-free DNA from these patients contains methylation fingerprints specifically related to the tumors. Furthermore, significant negative correlations between the methylation of *cytosine–phosphate–guanine dinucleotide* (CpG) and the expression levels of their putative target genes were discovered, concluding that these genes are epigenetically regulated by PitNET-specific differentially methylated probes [27].

A significant association between hypermethylation in PitNETs and the loss of neuronatin expression has been discovered in the last decade. It has been demonstrated that the co-localization of neuronatin to each major hormone-secreting cell type of the adenohypophysis and the decreased expression of this gene is associated with promoter hypermethylation, while the loss is irrespective of PitNET subtype. These findings suggest that epigenetic silencing of neuronatin has a significant impact on tumoral behavior and pathogenesis [28,29].

After acknowledging the main biomolecular aspects of PitNETs, a crucial question arises in the neurosurgeon’s mind: What is the connection between these genes and neurosurgery, and how can this information benefit the neurosurgeon? Although it may appear of no significant importance for the clinical practice at first glance, correlations between these two different scientific worlds have been concluded.

The most common PitNETs are represented by lactotrophinomas, followed in frequency by gonadotropinomas, somatotropinomas, corticotropinomas, and thyrotropinomas. However, sometimes no lineage differentiation, transcriptional factors, or hormone secretion can be identified, and in these cases, the tumors are considered null cell adenomas [30,31]. Similarly, tumors may produce unusual combinations of hormones and are known as plurihormonal adenomas [32].

### 2.1. Lactotrophinomas

Lactotroph PitNETs (LAs), also known as prolactinomas (PRLs), are the most frequently encountered functioning PitNETs in both men and women [33]. These common benign prolactin-secreting tumors derived from lactotrophs account for approximately 50% of all PitNETs, and between the second and fourth decades of life, the female-to-male ratio is up to 10:1, while after menopause, the ratio equalizes [33,34]. Microprolactinomas are very seldom proliferative and are of low concern for persistent long-term growth, being more frequent in women. Macroprolactinomas are very different and are more frequently seen in men [35]. These large tumors, although generally benign, are aggressive and invasive, often with extension into the suprasellar region and into the cavernous sinuses. In these cases, biochemical remission and tumor control can be achieved only with a multimodal therapeutic approach that includes neurosurgical excision, long-term treatment with dopamine agonists, and sometimes radiotherapy [35].

Various clinical and biological factors are associated with invasiveness and aggressiveness in LAs. Young patients, under 20 years old, tend to have tumors of larger dimensions and with a more aggressive behavior. At a young age, such a tumor should prompt exploration for a possible genetic predisposition, which is associated with a poorer prognosis [36]. Familial LAs comprise approximately 3% of the cases and are associated with genetic syndromes such as MEN1 and 4, familial isolated PitNET, Carney syndrome, and hereditary paraganglioma–pheochromocytoma syndrome [37,38]. Pediatric PitNETs report the germline *aryl hydrocarbon receptor-interacting protein (AIP)* gene mutation in approximately 20% of the cases [39,40], but also *menin (MEN1)* mutations, in both sporadic and familial cases [41,42]. Besides having more aggressive tumors, patients with AIP and MEN1 mutations tend to have cabergoline-resistant PRLs [3,43].

The tumoral dimensions also influence prolactin levels. Very high prolactin values have been correlated with poor surgical outcomes, while low levels may occasionally be observed in poorly differentiated tumors. However, low prolactin levels are also common in microprolactinomas due to the small tumor volume, and in large tumors, it is most often explained by stalk disconnection. Therefore, low prolactin levels alone should not be interpreted as a marker of poor differentiation [44,45].

Resistance to dopamine agonists has also been proven to be of major importance, as this is the first-line therapeutic option in LAs [46]. In cases with abnormal serum prolactin levels after dopamine agonist therapy, the resistant tumors exhibit a more severe clinical course, are larger in volume, more invasive, more aggressive, and have a higher Ki-67 index, especially in men [47,48].

It has been demonstrated that *the human pituitary tumor-transforming gene (PTTG)* is overexpressed in LAs with high invasiveness. This gene is located on chromosome 5, at position 5q33, and it induces cell transformation [49]. Thus, the PTTG abundance is a molecular marker for invasiveness in hormone-secreting PitNETs, and also plays a role in tumorigenesis [50]. It is worth mentioning that invasiveness refers primarily to radiological or intraoperative evidence of tumor extension into adjacent structures (e.g., cavernous sinus), while aggressiveness denotes biological behavior such as rapid growth, high Ki-67 index, resistance to medical therapy, or recurrence despite optimal treatment [51].

Expression of the *p53* tumor suppressor gene has been controversial in PitNETs due to technical problems. However, in some LAs, elevated prolactin expression and loss of p53 have been observed, potentially influencing tumoral behavior, promoting more aggressive characteristics [51].

Chromosome abnormalities were also involved in the clinical course of the disease, with loss of heterozygosity (LOH) being the most extensively studied. Invasive tumors demonstrated a significantly higher frequency of deletions affecting 11q13, 13q12-14, and 10q26. Moreover, allelic deletion correlates with increasingly invasive behavior [52]. Integrated genomic profiling reveals the loss of chromosome 11p, which impacts transcriptomic activity in aggressive PRLs. Comparison of genomic and transcriptomic data demonstrated that allelic loss impacted upon expression of genes located in the imbalanced region, among which DGKZ, CD44, TSG101, GTF2H1, and HTATIP2 were responsible for triggering aggressive and malignant phenotypes of PRLs [53].

Adhesion molecules and metalloproteases were shown to be involved in LAs. Expression of E-cadherin and beta-catenin was significantly lower in invasive PRLs, and the reduced expression was more frequently detected in invasive tumors. In addition, expression of E-cadherin was lower in macroprolactinomas, and decreased expression predicted higher Ki-67 indexes [54]. The matrix metalloproteinases inhibitor TIMP-2 was overexpressed in noninvasive PitNETs, suggesting that it could play a role in the aggressiveness of LAs [55,56,57].

Lastly, a variety of genes related to proliferation and invasion were described in LAs. Seven genes were associated with recurrence and progression: ADAMTS6, CRMP1, PTTG, ASK, CCNB1, AURKB, and CENPE, while ADAMTS6, CRMP1, ASK, CCNB1, and CENPE were also associated with tumor recurrence and progression [58].

Table 2 summarizes the correlations between biomolecular parameters and neurosurgical aspects in lactotropinomas.

### 2.2. Gonadotropinomas

Pituitary gonadotropinomas are usually classified as nonfunctioning PitNETs, since in the majority of cases they do not cause clinically evident hormone excess. However, approximately 35% secrete biologically active luteinizing hormone (LH) or follicle-stimulating hormone (FSH), in which case they are considered functioning gonadotropinomas [59]. These tumors are characterized by the expression of SF1, GATA3, and ERα, and often contain β-FSH or β-LH subunits. The vast majority of GAs are macroadenomas, mostly with supra—and/or parasellar extension. Usually, there is a hypersecretion of FSH, while the LH levels are normal or decreased. The hypothesis of the lack of biological activity in some cases with increased gonadotropin secretion is reported in the current literature [60].

Before 2017, most medical studies on nonfunctioning GAs were combined with null cell adenomas and other nonfunctioning PitNETs; thus, the data that specifically study GAs is rather scarce [61].

Newey et al. performed whole-exome sequencing using DNA from 7 nonfunctioning PitNETs originating from gonadotroph cells, and identified 24 mutations that occurred in independent genes with no recurrent mutations. The authors concluded that these tumors harbor few somatic mutations, consistent with their low proliferation rates and benign nature. However, it is worth mentioning that besides somatic mutations, other mechanisms are probably responsible for the etiology [62].

Falch et al. analyzed the gene expression of fast and slow-growing nonfunctioning GAs and found 350 genes that were significantly differentially expressed [63]. While *metadherin* *(MTDH)*, but not *endomucin (EMCN)*, demonstrated involvement in cell migration and association with epithelial-to-mesenchymal transition (EMT) markers, the study concluded that genes related to EMT have higher expression in fast-growing tumors. Furthermore, *MTDH* is an essential contributor to aggressiveness, while other genes might be useful as a biomarker tool for tumoral growth and possible therapeutic targets [63].

It has been stated that the noncoding RNA *Maternally Expressed Gene (MEG)*, which regulates p53 gene expression, is downregulated by methylation in a series of functioning GAs. This is especially of great interest since the downregulation of *MEG* promotes cellular proliferation. Notwithstanding, in GAs, the proliferation is counteracted by high expression of antiproliferative genes p27/p16 [64].

The Ki-67 expression is higher in patients who require a secondary surgical intervention, while in patients with a Ki-67 index lower than 3% a second surgery was less necessary. Furthermore, the proliferative index was also higher in those who received postoperative radiotherapy than in those treated only by neurosurgical intervention [65,66].

However, given their rarity, the full biomolecular mechanisms underlying the pathogenesis of functioning GAs are yet to be fully understood.

Regarding the neurosurgical approach, the transsphenoidal resection is the initial treatment of choice and can reduce endocrine disturbances, provide tissue for analysis, and improve neurologic manifestations [67]. Because the rate of recurrence in macroadenomas is high, the optimal surgical strategy is not yet defined, leading to high morbidity and mortality. In some cases, a flexible combination between the transsphenoidal and transcranial approach can maximize the grade of resection [68].

The correlations between the biomolecular aspects of GAs and neurosurgery are summarized in Table 3.

### 2.3. Somatotropinomas

Somatotroph PitNETs (SAs) comprise approximately 20% of all PitNETs and are well known for determining acromegaly, which leads to significant morbidity and mortality. Acromegaly is caused by dysregulated hypersecretion of growth hormone (GH), leading to an overproduction of insulin-like growth factor 1 (IGF-1) [69]. The most common tumor correlated with acromegaly is represented by densely granulated somatotroph adenoma (DGSA), usually highly hormonally active, in which the tumor cells express PIT1, GH, and alpha subunit of glycoprotein hormones (αSU) [70,71]. They are usually diagnosed at a younger age due to their hormonal activity and are smaller in size [70,71]. On the other hand, the sparsely granulated somatotroph tumor (SGSA) has fewer secretory granules and can be negative or only weakly positive for GH [72].

Many DGSA harbor activating mutations of the *GNAS* gene. The protein product of this gene, Gsα, mediates signaling from seven transmembrane domain G-protein coupled receptors to activate cyclic AMP, which explains the sensitivity to somatostatin inhibitors, unlike SGSAs, which are mainly resistant to medical therapy and respond better to surgical treatment [12].

Regarding the neurosurgical aspects, it has been demonstrated that SGSAs are much less likely to achieve remission after 3 months and during the follow-up period in comparison to DGSAs [73]. Furthermore, due to incomplete feasible resection, SGSAs are often reoperated, and approximately 14% achieve postoperative cure, in comparison to DGSAs with a percentage of 65%, independent of patients’ age and tumoral dimensions [73]. It is worth mentioning that in the preoperative settings, SGSAs tended to have higher Knosp grades, lower GH indexes, and normalized IGF-1 levels [74]. However, despite various therapeutic options, the neurosurgical approach is the main treatment, especially the endoscopic transsphenoidal approach, providing fewer complications and better outcomes [75].

In a recent study of 83 patients with SAs treated by the endoscopic transsphenoidal approach, the GH level at diagnosis and operation, tumor dimensions, and residual tumor were significantly correlated with remission results. In addition, patients with lower GH levels, smaller tumors, and no residual tumors were more likely to achieve biochemical remission. Similarly, patients with DGSAs histology were more likely to achieve GH levels less than 2.5 ng/mL than those with SGSAs [75].

A recent meta-analysis, including 1223 studies, was performed by Vuong et al. on the clinical and prognostic significance of granulation patterns in SAs [76]. The authors concluded that SGSAs had significantly larger tumoral dimensions at presentation, as the rate of macroadenomas was 3 times higher when compared to DGSAs. Furthermore, SGSAs had a significantly higher risk of cavernous sinus invasion and a higher Ki-67 index, which required a close postoperative radiologic follow-up. For tumors that needed postoperative somatostatin receptor ligand therapy and/or other medical therapy, DGSAs were correlated to a significantly better biochemical response rate. Regarding the biomolecular characteristics, it has been concluded that SGSAs were associated with a lower prevalence of the *GNAS* mutation [76].

Bakhtiar et al. analyzed the relationship between cytokeratin staining patterns and clinico-pathological features in SAs. They concluded that these tumors typically exhibit a perinuclear pattern (PP) and a dot pattern (DP) in cytokeratin immunostaining [77]. The authors stated that DP tumors had significantly larger dimensions and a higher Ki-67 proliferative index. Furthermore, the DGSAa typically exhibited a PP, while SGSAs exhibited a DP. The *GNAS* mutation was less frequent in DP tumors, and the frequency of DP was higher in younger patients. Given the more invasive and aggressive features of DP tumors, associated with diffuse growth and suprasellar extension, the endonasal approach could be limited, despite usually being reported as curative in more than 60% of SAs [78]. These characteristics could also be explained by a significantly higher expression of E-cadherin in DP tumors, which mainly influences tumoral growth and invasiveness [77].

The correlations between biomolecular, histopathological, and clinical aspects and neurosurgical implications in SAs are summarized in Table 4.

### 2.4. Corticotropinomas

Corticotroph PitNETs (CAs) account for approximately 15% of all PitNETs and cause ACTH oversecretion, responsible for Cushing’s disease [79]. This represents the most frequent cause of Cushing syndrome, or chronic excess of endogenous glucocorticoids. In 90% of the cases, it presents as a microadenoma, sometimes not visible on neuroimaging investigations [79]. In approximately 10% of the cases, CAs stain ATCH without causing Cushing disease and are considered silent (SCAs). These tumors are reportedly more aggressive, with increased cavernous sinus invasion and progression/recurrence [80].

From a histopathological perspective, CAs are categorized as densely granular, sparsely granular, and Crooke cell adenoma [81]. TPIT1 represents the transcriptional factor for CAs [82]. In these tumors, no common germline mutations were detected; however, somatic mutations were reported in up to 60% of the cases, and the most frequently encountered is an activating mutation in the *ubiquitin-specific peptidase 8 enzyme (USP8)*, which promotes tumorigenesis, leading to increased cell proliferation and invasiveness [64]. *USP8* mutations are strongly associated with microadenomas and are more frequent in women. The presence of these mutations may predict favorable responses to somatostatin analog pasireotide, which exhibits high affinity for SSTR5 and double SSTR2/SSTR5 positivity [83]. Moreover, in cases with *USP8* wild-type status, lower surgical cure rates, more invasive behavior, and poorer response to medical therapy were recorded. No significant difference in hormonal levels was observed concerning *USP8* status, but *USP8*-variant carriers were more likely to achieve surgical remission than wild-type PitNETs [83,84].

A recent study by Nerubenko et al. assessed the clinical significance of somatic *USP8* variants in CAs derived from patients with Cushing’s disease. The study concluded that in patients with CAs harboring *USP8* variants, there was a prevalence of microadenomas, a higher recurrence after successful surgery, and the prevalence of SST5 and SST2 receptors’ expression [85]. An unexpected finding of the study was positive SST2 expression in the majority of USP8-mutant CAs and in three of ten USP8 wild-type tumors [85]. Furthermore, the authors hypothesized that the *USP8* mutation may result in multidirectional alterations in the regulation of cell growth and proliferation, and hormonal production in CAs [85].

Similarly, *USP48* was also proposed as an essential factor in the pathogenesis of these tumors. Mutations in this gene were associated with female gender and smaller tumoral dimensions [86].

Kober et al. evaluated the expression of glucocorticoid (GR) and mineralocorticoid receptors (MR) in CAs and SCAs. They concluded that there was a correlation between the expression levels of *NR3C1* and *NR3C2*, the genes encoding these receptors. Consistent with prior data, the authors demonstrated a higher *NR3C1* expression in SCAs in comparison to tumors causing Cushing’s disease, but no difference in *NR3C2* was observed [87]. In addition, in patients with Cushing’s disease, both genes were negatively correlated with the tumoral size and morning plasma ACTH. Higher *NR3C2* expression was observed in patients with postoperative remission and in densely granular CAs, whereas the expression of both genes and GR protein was higher in *USP8*-mutated tumors. Finally, a negative correlation between GR and tumor size, and higher *NR3C1* expression was demonstrated in densely granulated tumors [87].

*TP53* mutations in CAs were also studied and were associated with a younger age, did not show sex predominance, and were more invasive, larger, with lower complete ressection rates. A Ki-67 index ≥ 3, together with p53 immunostaining and mitotic count, was correlated with tumor aggressiveness [88]. Uzilov et al. stated that *USP8* and *TP53* gene mutations are mutually exclusive, with the latter occurring only in *USP8* wild-type tumors [89]. The authors suggested that *USP8*-mutated tumors have better surgical remission rates, whereas *USP8* wild-type tumors, especially those with *TP53* mutations, are correlated with high invasiveness and worse clinical outcomes. Furthermore, *TP53* mutations were associated with larger tumoral volumes, higher proliferative indexes, higher Knosp grades, higher rates of subtotal resection, and lower 10-year survival rates in comparison to *USP8*-mutated or *USP8*/*TP53* wild-type tumors (27% versus 86–100%) [89,90].

Mutations in the *alpha thalassemia/intellectual disability syndrome X-linked (ATRX)* gene were also described in up to 32% of CAs, and were correlated with aggressiveness, resistance to therapy, and potentially metastatic PitNETs [91,92].

The management of CAs, especially with high aggressiveness and invasiveness, remains a therapeutic challenge due to incomplete resection. A multimodal approach could lead to gross total resection and biochemical remission in approximately half of the patients, and if remission is not achieved by neurosurgical intervention, other therapeutic options are required [93].

A summary of correlations between biomolecular aspects and neurosurgery in corticotropinomas has been presented in Table 5.

### 2.5. Thyrotropinomas

Tyrotroph PitNETs (TAs) account for less than 1% of all PitNETs, are characterized by excessive thyrotropin secretion, and are not linked to either germline or somatic mutations. The transcriptional factors for TAs are PIT1 and GATA3, and the hormones by immunohistochemistry are β-TSH and the α-subunit [37,61].

Although previous reports described the neurosurgical cure as difficult, given the invasive nature and larger tumoral dimensions, with the current ultrasensitive immunometric assays, these lesions are more often early diagnosed [94]. However, in those cases that were not early diagnosed, TAs are commonly invasive and large macroadenomas, and the first-line therapy is represented by transsphenoidal surgery, despite the mostly unsatisfactory results [95].

Although the biomolecular aspects of TAs have not been thoroughly studied, these tumors are known for the lack of expression of the *GNAS* oncogene [96,97]. The failure to detect evidence for activating mutations leaves open the search for alternative mechanisms underlying TA’s tumorigenesis [98].

From a genetic perspective, TAs are distinct from other PitNETs, as they do not have a hereditary background. A few cases were associated with multiple endocrine neoplasia type 1, which is linked to loss of heterozygosity on 11q13 and inactivating mutations of the *MEN1* gene, yet data suggest that menin does not play a causative role in the tumorigenesis of TAs [99].

It is worth mentioning that in the majority of cases, TAs overexpress PIT1, which is a lineage-defining transcription factor also associated with somatotropinomas and prolactinomas. Hence, it has been demonstrated that in approximately 70% of the cases, TAs are plurihormonal [100,101]. In addition, unlike other PitNETs, the Ki-67 proliferation marker is usually low in TAs, with sporadic exceptions [102], but the tumoral development and growth are often driven by differentiation rather than proliferation [94]. Moreover, despite old studies suggesting that TAs are negative for *p53*, a new study by Căpraru et al. demonstrated that expression of *p53* can be positive in up to 41% of these tumors [102]. The same research concludes that monohormonal tumors were larger than plurihormonal ones, but clinical and biological signs of hyperthyroidism were more frequent in the plurihormonal tumors. Similarly, monohormonal tumors had higher expression of *SSTR2A* and no level or low level of expression of *SSTR5*, while plurihormonal tumors (TSH+GH) expressed *SSTR5* at high levels [102]. Regarding the neurosurgical aspects, it has been stated that while transsphenoidal surgery allows complete resection in most cases, the curative rate oscillates between 60% and 75%, and is lower in cases of macroadenomas with sinus invasion [95]. The neurosurgical approach is also recommended in recurrences, although much more attention is needed due to tumor invasive regrowth and fibrosis, which can cause perioperative complications. The most significant predictor of surgical success remains the degree of invasion into the cavernous sinuses [103].

Currently, the criteria for a complete remission of TAs comprise the disappearance of hyperthyroidism and neurological manifestations, the removal of the entire tumor demonstrated on neuroimaging studies, and normal TSH, FT3, and FT4 in the blood for at least 3 months postoperatively [104].

The correlation between TAs’ biomolecular aspects and neurosurgical implications has been summarized in Table 6.

### 2.6. Null Cell Adenomas

Null cell adenomas (NcAs) represent approximately 0.6% of all PitNETs and are defined as immunonegative for all adenohypophyseal hormones, with a lack of cell-type-specific transcription factors [30]. Before 2017, NcAs were considered nonfunctioning, given the lack of hormone expression. Lee et al. analyzed 147 PitNETs that were previously classified as NcAs, and concluded that only 68 cases were potentially correctly diagnosed. The authors concluded that PIT1 can be utilized as a second-tier immunostain in cases of clinically nonfunctioning PitNETs that are immunonegative for all hormones and SF1, to segregate rare cases of PIT1-positive PitNETs from NcAs [105]. Thus, it is of significant importance to note that, due to recent changes in the pathological classification of these tumors, there is a scarcity of data regarding their biomolecular aspects. Hence, the current review of NcAs is mainly based on the current state of knowledge.

A recent study by Woo et al., using the 5th WHO Classification, the latest version, concluded that patients with NcAs and multiple PitNETs had less disease-free survival compared to those with GA. In addition, consistent with prior studies, the authors demonstrated that these tumors were a more aggressive subtype that was prone to invade, with a higher risk of residual disease [106].

Balogun et al. analyzed 31 patients with NcAs and concluded that the preoperative invasion into the cavernous sinus was a predictor of tumoral remnants postoperatively [107]. Moreover, preoperative invasiveness into the cavernous sinus and negative *P27* expression independently predicted subsequent growth of the residual tumor. In the same study, when compared to patients with sparsely granulated adenomas in which tumors grew rapidly postoperatively and slowly after surgery, those with NcAs had tumors that grew rapidly prior to surgery and continued to exhibit rapid postoperative growth. When it comes to the Ki-67 index, NcAs had higher values, over 3%, which was consistent with prior studies [107,108,109]. A summary of the link between biomolecular and neurosurgical aspects is presented in Table 7.

### 2.7. Plurihormonal Adenomas (PAs)

PAs are categorized based on their transcription factor expression in PIT1-positive PitNETs, and with more than one transcription factor (PAwUIC) [61]. They are characterized by aggressive behavior with higher values of the percentage of invasiveness into neighboring anatomical structures. The PAs can only be identified by pathological assessment, and an initial endocrinologic diagnosis according to hormonal and clinical features is necessary [110]. A study by Aydin et al. evaluated data from 27 patients with PAs and concluded that the majority were positive for more than one transcription factor. In comparison, nine patients were diagnosed with PIT1-positive PAs. More than 80% of the patients included in the study had macroadenomas, and high aggressivity was recorded in nearly half of these cases after pathological examination. Approximately 77% of the patients with PAs with more than one transcription factor had features of non-functioning tumors, and four patients had features of functioning PitNETs. Concerning the neurosurgical aspects, PAwUIC showed a lower rate of gross total resection, while in PIT1-positive PAs, a rate of more than 77% was registered [110].

A study by Micko et al. compared the clinicopathological parameters of PAwUIC with transcription factors for gonadotropinoma (TFGA) expression with gonadotropinomas that only express TFGA [111]. The results concluded larger tumor dimensions in the TFGA-only group. Regarding the neurosurgical aspects, in TGFA-plus patients, invasive behavior was more frequent. Furthermore, in the TFGA-only group, gross-total resection was significantly higher in comparison to the non-functioning TFGA-plus group. These findings suggest that TFGA-positive tumors are more aggressive when an additional transcription factor is expressed. Moreover, the study concluded that in these lesions, shorter radiographic surveillance and earlier reintervention are recommended [111].

PIT1-positive plurihormonal adenomas were also described as very distinctive entities. They are mostly reported as macroadenomas with aggressive behavior, very invasive, and with a higher rate of recurrence [112]. These tumors are not always silent and represent poorly differentiated monomorphous plurihormonal PIT1 lineage PitNETs [113]. The only neurosurgical approach that can offer the patient the most benefits is aggressive neurosurgical intervention. Very often, due to tumor persistence, external beam radiation is required. Nevertheless, aggressive surveillance must be initiated immediately after the diagnosis, with a low threshold for radiotherapeutic intervention, even in cases of minimal recurrent diseases [112].

A summary of the link between biomolecular parameters and neurosurgical aspects in PAs is presented in Table 8.

A synthesis table that integrates the significant biomolecular markers across all PitNET subtypes with their direct neurosurgical implications is presented below (Table 9).

### 2.8. Postsurgical Clinical Implications and Decision-Making

An important aspect regarding advances in the molecular and histopathological fields is that all the presented biomarkers have a direct influence on intraoperative management and postoperative planning in PitNETs. Notwithstanding that the majority of these molecular and genetic biomarkers are identified after the neurosurgical intervention, their clinical relevance extends to the multidisciplinary management [70].

One of the most essential studied biomarkers in PitNETs remains the Ki-67 proliferative index, which indicates not only a higher proliferative tumoral feature but also a greater risk of recurrence. Furthermore, a higher Ki-67 index in neurosurgical practice translates to lower chances of achieving maximal resection. In addition, in the postoperative setting, a higher Ki-67 index indicates the need for a more rigorous MRI follow-up and, in some cases, the necessity of early radiotherapy adjuvant [114].

Similarly, the PTTG plays a significant role in postsurgical clinical implications and decision-making in PitNETs. This biomarker, which has an oncogenic role, is responsible for local invasion, especially in lactotroph tumors. Its overexpression is associated with an increased risk for cavernous sinus invasion, which will limit the extent of resection [115].

Similarly, p53 mutations or overexpression have been associated with a more aggressive tumor behavior in lactotroph and corticotroph PitNETs as a sign of impending recurrence after operation [97].

Cell adhesion marker changes, such as E-cadherin and β-catenin, are equally significant. Their loss suggests tumor invasiveness, which neurosurgeons can anticipate as a predictor of cavernous sinus extension and reduced likelihood of gross total resection. In these situations, long-term radiological follow-up is necessary, with adjuvant therapy being a consideration in cases of infiltrative growth [12].

Matrix metalloproteinase regulators also contribute to the invasive characteristics of the lactotroph tumor. Low TIMP-2 levels are associated with increased extracellular matrix degradation, greater surgical complexity, and a higher risk of incomplete resection. Such patients need close observation for recurrence after surgery and could be offered early adjunctive treatment [116].

Genetically, the GNAS mutations are particularly relevant in somatotroph PitNETs. Densely granular somatotroph adenomas with GNAS mutations are likely to be responsive to somatostatin analogs and tend to be less challenging to resect. However, sparsely granular variants are less responsive and are more likely to require multimodal therapy and closer follow-up. Additionally, the cytokeratin pattern (dot-like versus perinuclear) can provide insight into tumor morphology and behavior. The DP-pattern somatotroph tumors are generally larger, more invasive, and less likely to be totally resected, and thus are candidates for early consideration of radiotherapy or reoperation [117,118].

In corticotrophinomas, *USP8* mutations are associated with smaller, less malignant microadenomas and higher rates of surgical remission. The same mutations also predict improved response to medical treatment such as pasireotide, which impacts endocrine follow-up management [83]. *TP53* and *ATRX* mutations, on the other hand, are associated with tumors with a more malignant, therapy-resistant profile, where subtotal resection and early adjuvant radiotherapy or medical treatment are typically required [79,88].

Genetic syndromes such as AIP and MEN1 germline mutations are of surgical significance since they are invariably associated with large, dopamine-resistant tumors occurring in young patients. These are often so severe that they necessitate early surgery despite medical treatment, followed by genetic counseling and long-term follow-up [119].

Finally, definitions of invasiveness and aggressiveness deserve special attention. Invasiveness refers to the radiologic or intraoperative extension of the tumor into adjacent structures such as the cavernous sinus, sphenoid sinus, or suprasellar region, which signifies reduced potential for gross total resection and can necessitate a multimodal surgical approach. Aggressiveness, on the other hand, characterizes the biological behavior of tumors, as exhibited by rapid growth, an elevated Ki-67 index, resistance to treatment, or early recurrence, and directly determines postoperative treatment, specifically the indication for adjuvant radiotherapy and personalized follow-up.

Integrating these findings into postoperative decision-making enhances the precision of patient management and optimizes long-term outcomes. To provide a practical framework for clinical use, Table 10 summarizes the main biomolecular markers and concepts in PitNETs, linking them directly to neurosurgical and postsurgical decision-making.

## 3. Conclusions

The biomolecular mechanisms underlying the pathogenesis of PitNETs have become a significant area of interest in the last decade, especially since the 5th Edition of the World Health Organization Classification of Endocrine and Neuroendocrine Tumors. Although at first glance the biomolecular world may appear of no significant interest to the neurosurgeon, essential correlations between these two scientific worlds were concluded. By understanding the complex mechanisms and molecular drivers behind tumorigenesis, neurosurgeons may be able to predict prognosis and guide postoperative therapeutic management and follow-up intervals, collaborating with other specialists.

Although biomolecular research in PitNETs is currently in its infancy, it will likely help broaden the understanding of this pathology. Future research should explore the integration of molecular profiling into surgical planning, as well as into the development of personalized follow-up strategies and the selection of adjuvant therapies driven by researched biomarkers.

Ultimately, bringing biomolecular discoveries into everyday clinical care will depend on strong collaboration between neurosurgeons, endocrinologists, pathologists, and molecular scientists. Such teamwork is essential for translating research findings into practical strategies and providing patients with genuinely personalized treatment for PitNETs.

## Figures and Tables

**Table 1:** The key transcription factors in PitNETs are represented by PIT1—Pituitary Transcription Factor 1; SF1—Steroidogenic Factor 1; TPIT—T-box Pituitary Transcription Factor.

Transcription Factor	
Pituitary Cell Lineage	Associated Tumor Types
PIT1	
Somatotrophs, Lactotrophs, Thyrotrophs	GH-, PRL-, TSH-secreting tumors
**SF1**	
Gonadotrophs	Non-functioning PitNETs
**TPIT**	
Corticotrophs	ACTH-secreting tumors (e.g., Cushing’s disease)
**Negative for all**	
Null cell lineage (no lineage-specific factor)	Rare; often reclassified
**Multiple (including PIT1, SF1, TPIT)**	
Multiple pituitary lineages (e.g., somatotroph + corticotroph)	Plurihormonal tumors with more than one transcription factor (PAwUIC)

**Table 2:** Correlations between biomolecular parameters and neurosurgical aspects in lactotropinomas.

Biomolecular/Genetic Parameter	Associated Tumor Behavior/Marker	Neurosurgical Implication
Tumor size and prolactin level correlation	High PRL correlates with large tumor size; low PRL may suggest poor differentiation or stalk effect	Larger tumors are more complex to resect completely; low PRL in large tumors warrants differential diagnosis
Gender and age	Males and younger patients (<20) show more aggressive, larger tumors	Often present as macroadenomas with invasive features, requiring multimodal therapy
AIP/MEN1 mutations	Associated with familial PRLs, younger onset, high recurrence, and cabergoline resistance	Poor response to dopamine agonists → surgery more often needed; larger, more invasive tumors
Cabergoline resistance	Linked to AIP/MEN1 mutations; resistance associated with high Ki-67, invasiveness	Often necessitates transsphenoidal surgery due to failed medical therapy
High Ki-67 index	Proliferation marker; associated with larger size, dopamine agonists resistance, and aggressiveness	Predicts less favorable resection outcomes; requires long-term radiological monitoring
PTTG overexpression	Promotes invasiveness and tumorigenesis	May correlate with higher surgical complexity and increased recurrence risk
p53 inactivation/loss	Found in some aggressive LAs with high PRL expression	Potential marker for aggressive surgical course; limited practical use currently
Chromosomal deletions (LOH: 11q13, 13q12-14, 10q26)	Associated with invasiveness and poor prognosis	May reflect deeper infiltration and the probability of subtotal resection
11p loss (genes: DGKZ, CD44, TSG101, etc.)	Linked to aggressive molecular phenotypes	Suggests poor tumor containment and risk of postoperative regrowth
Low E-cadherin/ beta-catenin expression	Impairs cell adhesion; correlates with invasiveness and high Ki-67	Indicates a higher likelihood of extrasellar or cavernous sinus extension
Low TIMP-2 expression	TIMP-2 inhibits metalloproteinases; low expression promotes tissue invasion	Suggests infiltrative borders and is less favorable for complete resection
Proliferation genes (ADAMTS6, CRMP1, CCNB1, etc.)	Associated with recurrence and progression	Molecular profiling could identify patients at higher risk of incomplete resection or regrowth post-op

**Table 3:** Correlation between biomolecular aspects and neurosurgery in gonadotropinomas.

Biomolecular/Histological Feature	Neurosurgical Aspect/Behavior
SF-1, GATA3, ERα, β-FSH/LH expression	Defines the gonadotroph lineage; tumors tend to be macroadenomas, often invasive (cavernous/suprasellar), which lowers the chances of GTR.
Low somatic mutation burden	Indicates indolent cellular behavior; however, invasiveness is often anatomical rather than genetic, complicating complete resection.
EMT-related gene overexpression (e.g., *MTDH*)	Correlates with fast-growing (aggressive tumors), higher invasiveness, and risk of residual tumor post-surgery.
Ki-67 > 3%	Associated with higher recurrence/reintervention rates, especially when residual tumor remains.
Residual tumor/invasion (suprasellar/cavernous)	Lower rates of GTR and higher recurrence risk, regardless of low proliferative index.

**Table 4:** Correlations between biomolecular, histopathological, and clinical aspects, as well as neurosurgical implications, in SAs.

Biomolecular Parameter	Histological Subtype/Marker	Neurosurgical Aspect	Clinical Implication
**Granulation** **pattern**	Densely granulated somatotroph adenoma (DGSA)	Smaller, well-defined tumors	Higher rate of gross total resection (GTR); lower recurrence risk
	Sparsely granulated somatotroph adenoma (SGSA)	Larger, invasive, irregular margins, aggressive behavior	Lower resectability; subtotal resection is more likely
**Ki-67 index**	Higher in SGSA	Correlates with invasiveness and early regrowth	Requires closer postoperative radiologic follow-up
**Cytokeratin** **pattern**	Dot-like pattern in SGSA	Associated with diffuse growth and suprasellar extension	May limit the endonasal approach; higher chance of incomplete resection
**Hormonal** **profile**	High GH and IGF-1 levels in SGSA	Correlates with tumor volume and surgical complexity	Suggests higher biological activity and tumor aggressiveness
**Transcription factors** **(e.g., PIT1)**	Common to both subtypes	Define lineage, not invasiveness directly	Help classify tumor subtype, but less predictive of surgical outcome
**Mixed GH/PRL expression**	Frequently in DGSA	No distinct surgical implications if not invasive	May affect postoperative hormonal management
**Somatostatin** **receptor** **expression (SSTR2)**	Higher in DGSA	Not a surgical parameter per se	Better response to SSA if surgery is incomplete
***GNAS*** **mutation prevalence**	Lower in SGSAs and higher in DGSAs	Lower *GNAS* mutation prevalence suggests that medical treatment is not indicated (tumor resistance) and the surgical treatment is, ore indicated	Higher *GNAS* mutation prevalence in DGSAs suggests that tumor responds better to medical therapy

**Table 5:** Summary of correlations between biomolecular aspects and neurosurgery in corticotropinomas.

Biomolecular Feature	Neurosurgical Correlation	Notes
***USP8*** **mutations**	Associated with microadenomas, higher surgical remission, and less invasive behavior	More frequent in women; high expression of SST5/SST2 predicts better response to pasireotide
***USP8*** **wild-type**	Larger, more invasive, lower surgical cure rate, poorer response to medical therapy	Often carries *TP53* mutations; requires more aggressive surgery
***TP53*** **mutations**	Highly invasive, lower complete resection rate, larger size, higher Knosp grade	It occurs only in *USP8*-WT tumors; it is associated with low 10-year survival (27%)
***USP48*** **mutations**	Correlated with smaller tumors, more frequent in females	Less aggressive subtype; clinical implications still under study
***SST2*** **and *SST5* expression**	May predict response to somatostatin analogs and relate to tumor control post-surgery	Higher SST expression seen in *USP8*-mutant tumors
***NR3C1*** **(glucocorticoid receptor) expression**	Negatively correlated with tumor size and ACTH levels	Higher expression in dG-CAs and *USP8*-mutant tumors; possible surgical prognostic marker
***NR3C2*** **(mineralocorticoid receptor)**	Higher expression in post-op remission cases, especially in dG-CAs	Could aid post-surgical outcome predictions
**GR (glucocorticoid receptor)**	Higher in smaller tumors, associated with surgical success	Correlates with *USP8* mutation and lower invasiveness
**Histological subtype: densely granulated (dG)**	Better prognosis, smaller tumors, and more remission post-surgery	Associated with higher *NR3C1*/*NR3C2* expression
**Histological subtype: sparsely granulated (sG) or Crooke cell**	Often larger and more invasive, associated with worse surgical outcomes	More likely to be *USP8*-WT and resistant to therapy
**Silent corticotroph PitNETs (SCAs)**	Tend to be larger, more invasive, more recurrent, harder to detect early	Often not biochemically active; detected via imaging or mass effect
***ATRX*** **mutations**	Linked to aggressiveness, resistance to therapy, and potential for metastasis	Found in up to 32% of aggressive CAs

**Table 6:** Summary of the link between biomolecular aspects and neurosurgery in thyrotropinomas.

Molecular/Histological Feature	Implication for Surgery or Neurosurgical Outcomes
Overexpression of PIT-1, plurihormonality (TSH + GH/PRL)	Large macroadenomas with more invasive behavior; lower GTR rates due to cavernous sinus or suprasellar extension
Low Ki-67 index (<3%)	Slow-growing at the cellular level, but size/invasiveness is often driven by differentiation, not by proliferation
Somatostatin receptor expression	*SSTR2A* is predominant in monohormonal cases, *SSTR5* is predominant in plurihormonal cases; it helps in postoperative treatment planning when residual tumor remains

**Table 7:** A summary of the link between biomolecular and neurosurgical aspects in null cell adenomas.

Biomolecular Feature/ Biomarker	Neurosurgical/Clinical Implications
Lack of hormones transcription factors	Defines true null cell subtype; associated with more aggressive behavior and lower disease-free survival
Cavernous sinus invasion	Predicts lower rates of gross total resection and a greater risk of residual tumor post-op
MIB-1 (Ki-67 index) > 3%	Higher proliferation, correlates with aggressive behavior and poorer progression-free survival
Negative *P27* expression	Marker of increased risk of postoperative regrowth, potentially guiding follow-up strategies
True NcA rarity via epigenomics	Most “NCAs” may actually be misclassified gonadotroph or corticotroph tumors, complicating prior prognostic assumptions

**Table 8:** Summary table linking neurosurgical aspects and biomolecular parameters in plurihormonal adenomas; EBRT—External beam radiation therapy.

Subtype/Biomolecular Profile	Transcription Factors	Tumor Size	Aggressiveness	Gross Total Resection (GTR)
	Hormonal Function		Invasiveness	Neurosurgical/Management Notes
**PIT1-positive PAs**	PIT1 only	Mostly macroadenomas	Moderate to high	**>77%**
	~50% functioning		Present in many cases	Better resectability than PAwUIC
**PAwUIC** (PAs with more than one TF)	Multiple (incl. PIT1, SF1, TPIT)	Mostly macroadenomas	High (≈50% cases)	Lower than PIT1+ PAs
	Mostly non-functioning (77%)		Higher % of invasiveness	Poorer surgical outcomes, early recurrence risk
**TFGA-only gonadotropinomas**	TFGA only	Larger tumor size	Moderate	Significantly higher than TFGA-plus
	Often non-functioning		Less invasive	More favorable surgical outcome
**TFGA-plus tumors**	TFGA + another TF	Smaller than TFGA-only	Higher than TFGA-only	Lower GTR
	Often non-functioning		More invasive	Shorter surveillance intervals, early reintervention needed
**Plurihormonal PIT1+ adenomas**	PIT1 with multiple hormone expression	Mostly macroadenomas	Very high (aggressive, recurrent)	Often incomplete due to invasiveness
	Not always silent (some functioning)		Very invasive	Requires aggressive surgery + EBRT + close follow-up

**Table 9:** Biomolecular markers with direct neurosurgical relevance in PitNETs.

Biomolecular/Genetic Marker	Subtype	Tumor Behavior	Neurosurgical Relevance
**Ki-67 index**	All PitNET subtypes	High Ki-67 (>3%) is associated with invasiveness, early regrowth, and poor prognosis	Predicts lower gross total resection (GTR), need for closer radiologic follow-up
***PTTG* (Pituitary Tumor Transforming Gene)**	Lactotroph, others	Promotes proliferation, invasion, and tumorigenesis	Overexpression = higher surgical complexity, increased recurrence risk
**p53 abnormalities**	Lactotroph, corticotroph	Loss of function or mutations linked to aggressive phenotypes	A marker of poor surgical outcomes, higher recurrence, and limited current utility
**E-cadherin/β-catenin**	Lactotroph, somatotroph	Low expression = impaired adhesion, invasiveness	Predicts cavernous sinus invasion, subtotal resection probability
**TIMP-2 (Tissue inhibitor of metalloproteinases)**	Lactotroph	Low expression linked to invasive growth	Suggests infiltrative borders, reduced likelihood of complete resection
***GNAS* mutations**	Somatotroph (DGSA)	Activating mutation → responsiveness to somatostatin analogs	DGSA: smaller tumors, higher GTR, better prognosis; SGSA: resistant, harder resection
**Cytokeratin pattern (PP vs** **. DP)**	Somatotroph	Dot pattern = larger, more invasive tumors	DP tumors often require reoperation; limits endonasal approach effectiveness
***USP8* mutations**	Corticotroph	Microadenomas, less invasive and have better remission	Predicts higher surgical cure rates, pasireotide sensitivity
***TP53* mutations**	Corticotroph (USP8-WT)	Large, invasive, high Knosp grade, poor survival	Lower GTR, higher recurrence → aggressive surgical strategy needed
***ATRX* mutations**	Corticotroph (aggressive/rare)	Associated with therapy resistance, potential for metastasis	Predicts poor surgical prognosis, may justify early multimodal therapy
***NR3C1*/*NR3C2* (glucocorticoid & mineralocorticoid receptor)**	Corticotroph	Expression correlates with remission and smaller tumor size	Possible prognostic markers for surgical outcome
**Plurihormonality (multiple TF expression)**	PIT1+, PAwUIC	Larger, invasive macroadenomas	Associated with lower GTR, early recurrence → closer follow-up required
**AIP/MEN1 germline mutations**	Lactotroph (familial, pediatric)	Larger, more aggressive, cabergoline resistance	Often need surgery despite medical therapy; high recurrence risk

**Table 10:** Biomolecular markers in PitNETs: neurosurgical relevance, postsurgical implications, and definitions of invasiveness/aggressiveness.

Marker/Concept	Tumor Subtype(s)	Findings/Biological Role	Practical Implications for Neurosurgeons	Postsurgical Decision-Making
**Ki-67 index**	All PitNETs	High Ki-67 (>3%) = proliferative activity, recurrence risk	Anticipate a higher risk of incomplete resection, and counsel the patient about the prognosis	Guides the intensity of follow-up MRI; may justify early radiotherapy if very high
**PTTG (Pituitary Tumor Transforming Gene)**	Lactotroph, others	Oncogenic role: promotes invasion	Anticipate increased risk of cavernous sinus invasion	Identifies patients at higher risk of recurrence → closer follow-up
**p53 abnormalities**	Lactotroph, corticotroph	Mutations/overexpression linked to aggressive tumors	Consider a higher recurrence probability after surgery	May justify adjuvant radiotherapy or inclusion in clinical trials
**E-cadherin/β-catenin**	Lactotroph, somatotroph	Loss of adhesion molecules = invasiveness	Anticipate cavernous sinus invasion, lower chance of GTR	Radiological follow-up for progression; adjuvant therapy in infiltrative cases
**TIMP-2**	Lactotroph	Low levels → matrix degradation, invasiveness	Surgical difficulty, incomplete resection are more likely	Post-op recurrence monitoring; adjuvant therapies considered
***GNAS* mutation**	Somatotroph	DGSA is more responsive to SSA, SGSA resistant	DGSA—easier to resect, better prognosis	SGSA may require multimodal therapy, closer follow-up
**Cytokeratin pattern (PP vs** **. DP)**	Somatotroph	DP = larger, invasive tumors	DP tumors—anticipate subtotal resection	Early consideration for radiotherapy/reoperation
***USP8* mutation**	Corticotroph	Smaller, less invasive microadenomas	Higher chance of surgical remission	Predicts sensitivity to pasireotide; better follow-up prognosis
***TP53*, *ATRX* mutations**	Corticotroph	Associated with aggressive, therapy-resistant tumors	Anticipate subtotal resection, high recurrence	Justify adjuvant radiotherapy or medical therapy early
**AIP/MEN1 germline mutations**	Pediatric/ familial PitNETs	Large, aggressive, dopamine-resistant	Likely surgical indication despite medical therapy	Genetic counseling, close long-term follow-up
**Invasiveness** (definition)	All	Radiological/surgical extension into adjacent structures (e.g., cavernous sinus, sphenoid, suprasellar)	Predicts a lower chance of GTR	Determines need for repeat surgery or multimodal approach
**Aggressiveness** (definition)	All	Biological behavior: rapid growth, high Ki-67, recurrence, resistance to therapy	Alerts the surgeon about a higher recurrence risk	Guides adjuvant radiotherapy and personalized follow-up

## Data Availability

The data presented in this study are available on request from the corresponding author.
